# The effects of interval training on peripheral brain derived neurotrophic factor (BDNF) in young adults: a systematic review and meta-analysis

**DOI:** 10.1038/s41598-021-88496-x

**Published:** 2021-04-26

**Authors:** Patricia Concepción García-Suárez, Iván Rentería, Eric P. Plaisance, José Moncada-Jiménez, Alberto Jiménez-Maldonado

**Affiliations:** 1 Facultad de Deportes Campus Ensenada, Universidad Autónoma de Baja California, 22890 Ensenada, Baja California México; 2 Department of Human Studies, University of Alabama at Birmingham, Birmingham, 35294 USA; 3 Human Movement Sciences Research Center, University of Costa Rica, San José, 1200 Costa Rica

**Keywords:** Neuroscience, Physiology

## Abstract

The aim of the current meta-analysis was to determine the effects of acute and chronic interval training (IT) on serum and plasma BDNF concentrations in healthy young adults. A literature search was performed using six databases until February 2020. The TESTEX scale was used to assess the quality of studies. Effect sizes (ES) were computed and two-tailed α values < 0.05 and non-overlapping 95% confidence intervals (95% CI) were considered statistically significant. Heterogeneity, inconsistency (*I*^2^), and small-study effects using the Luis Furuya–Kanamori (LFK) index were examined. Fifteen studies (n = 277 participants, age = 24 ± 3 years) were included. The overall effects of IT on circulating BDNF concentrations were moderate and significant (ES = 0.62, 95% CI 0.00, 1.24, heterogeneous (*p* < 0.001), highly inconsistent (*I*^2^ = 90%), and with major asymmetry (LFK index = 2.76). The acute effect of IT on peripheral BDNF levels was large and significant (ES = 1.10, 95% CI 0.07, 2.14), heterogeneous (*p* < 0.001), highly inconsistent (*I*^2^ = 92%), and with major asymmetry (LFK index = 3.34). The chronic effect of IT on circulating BDNF was large and significant (ES = 0.93, 95% CI 0.40, 1.46), heterogeneous (*p* < 0.001), with moderate inconsistency (*I*^2^ = 70%), and minor asymmetry (LFK index = 1.21). Acute and chronic IT elicited a moderate increase in serum and plasma BDNF concentrations in a healthy young population.

## Introduction

Brain-derived neurotrophic factor (BDNF) was discovered in the early 1980s^1^ and belongs to the neurotrophin family of proteins^2^. Early studies in rodents showed an association between BDNF and synaptic plasticity, neuronal growth, neuronal survival, and cognitive processes^3–6^. BDNF binds to a specific tyrosine kinase receptor which induces TrkB tyrosine phosphorylation and activation in its cytoplasmic and kinase domains BDNF-brain-TrkB. The kinase domain recruits and activates specific proteins in the cytoplasm to activate signaling pathways that regulate cognition and synaptic plasticity^7,8^.

Although different cell types synthesize and release BDNF (e.g. adipocytes, skeletal muscle, immune cells, vascular endothelial cells, among others)^9–11^, the hippocampus of the brain is considered the main source of BDNF in mammals^12–14^. Interestingly, reports indicate that this neurotrophin can cross the blood–brain barrier^15^ and that peripheral circulating BDNF concentrations are associated with brain function^16–18^. In agreement, studies in humans demonstrate that peripheral BDNF concentrations are positively associated with hippocampus size and cognitive performance^19,20^, inversely associated with mood disorders^21–23^. Likewise, data suggest that BDNF has anti-inflammatory effects on brain in patients with Parkinson’s disease^24^.

Experimental studies, narrative reviews, and meta-analysis have indicated that aerobic exercise (moderate-intensity continuous training-MICT) increases circulating BDNF concentrations and improves brain function^25–27^. Thus, MICT is considered an effective strategy to induce neuroprotection^28^ and to improved brain function. Despite the many benefits of exercise, perceived or real “lack of time” is reported most frequently as the primary barrier that most individuals around developed or developing countries do not exercise regularly^29,30^.

Interval training (IT) modalities can be performed in a fraction of the time as MICT and have been shown to produce similar improvements in peripheral metabolism compared to MICT^31–33^. However, the latter effect is dependent on the population studied. Specifically, in untrained and patients with coronary artery disease, MICT may produce superior results than IT^34–36^. By its very nature, IT represents a potential solution for individuals that report “lack of time” as a barrier to exercise participation. IT consists of repeated short and long bouts of high-intensity exercise (near maximal, maximal, or supramaximal intensity -maximal heart rate, VO_2_max, peak power output, superior to maximal lactate state-state velocity). High-intensity bouts of exercise are interspersed with recovery periods (light-moderate exercise intensity or passive recovery), with a full-session of IT requiring ≤ 30 min to be completed^37,38^. Therefore, IT can be easily adapted to participants representing different ages, fitness levels, or health status. Although there is considerable variation and classifications in the literature, high-intensity interval training (HIIT) and sprint interval training (SIT) are the primary forms of IT reported. HIIT is characterized by near maximal bouts of exercise ranging from 2–4 min per interval With SIT, bouts are shorter in length (~ 30 s), but are maximal or supramaximal^39,40^ (See Table 1).

**Table 1:** Studies included in the meta-analysis.

Reference	Sample size (male/female)	Fitness	Age (yr.) M ± SD	BDNF collection	Exercise characteristics (duration/frequency/mode)	Protocol	HIIT classification (by Wen et al. 2019)	Main findings	TESTEX score
Cabral-Santos et al. 2016	10 (10/0)	Physically active	25.2 ± 1.7	Serum	Crossover acute high-intensity exercise/motorized treadmill	High (2.5 km) and low (1.25 km) session at 1:1 min VO_2max_ velocity with passive recovery	Acute moderate-volume and moderate-interval	Both protocols increased BDNF concentrations. Nonetheless, the BDNF response was not dependent of exercise volume	7
DiBattista et al. 2018	11 (11/0)	Physically active	28.8 ± 5.3	Plasma	Acute and after 2 weeks/three times a week/cycle-ergometer	3-min warm-up at 50 W, 8 × 60 s intervals in sessions 1–2, 10 × 60 s intervals in sessions 3 – 4, 12 × 60 s intervals in sessions 5 – 6 at W_peak_ interspersed 75 s of active recovery and 3 min cool-down at 50 W	Short-term, moderate-volume and moderate-interval HIIT	Both first and last session increased BDNF concentrations immediately after the exercise session, ST-HIIT did not change BDNF levels at baseline	6
Figuereido et al. 2019	11 (11/0)	Physically active	22.5 ± 5.4	Serum	Acute and eight week intervention/motorized treadmill	5-min warm-up at 50% VO_2max_ speed, 60 s 100% sVO_2max_ with 60 s passive recovery (no exercise) until completion of 5 km	Acute, high-volume, moderate-interval HIIE	BDNF levels increased after the session completion compared at baseline	5
Gmiat et al. 2017	14 (0/14)	Sedentary	30.8 ± 18.6	Serum	Acute high intensity exercise/whole body circuit	3 × 30 s of AMRAP of 10 circuit whole body exercises (i.e., jumping jacks, push-ups, abdominal crunch, squat, plank, triceps dips, high knees/running, lunges, push-up with rotations and side plank) with 2-min recovery between sets	Acute, high-volume, short-interval HIIE	noted decrease of BDNF after 1 h HIIE was present in both young and middle age participants	7
Heibisz et al. 2018	26 (17/9)	Sportsmen	19.2 ± 5.2	Serum	6 months/2 – 3 sessions a week/cycle-ergometer	HIIT: 5 to 7 5-min bouts at 85 – 95% P_max_ with 12-min of moderate activity efforts at 55 – 60% P_max_ 2 × a week SIT: 3 × 3 to 4 30 s bouts at all-out effort with 90 s of active recovery at 50 W and 25-min of low effort (45 – 55% Pmax) between sets	HIIT: Long-term, high-volume, Long-interval training SIT: Long-term, moderate-volume, SIT	Decreases of BDNF concentrations 10 and 60 min after sprint test in the SIT group after 2 and 6 month intervention	11
Heisz et al. 2017	66 (24/42)	Sedentary	20.7 ± 2.8	Serum	6 weeks/~ 3 times a week/cycle-ergometer	3-min warm-up at 50 W, 10 × 60 s of high intensity bouts performed at ~ 80%W_max_/~ 85 – 95% HR_peak_ combined with 60 s active recovery at ~ 30%W_max_ and 2-min cool-down at 50 W	Mid-term, moderate-volume, moderate-interval HIIT	BDNF did not significantly change from the intervention, however individual differences between low and high responders to exercise are directly related to the increase of BDNF concentrations	11
Kujach et al. 2019	36 (36/0)	Physically active	21.3 ± 1.3	Serum	Acute/cycle-ergometer	5-min warm-up at 1.5 W/kg body mass followed by 6 × 30 s all-out bout with fly-wheel at 0.075 kg/kg with resting periods of 4.5-min	Acute, low-volume, SIT	Increase of peripheral BDNF in experimental group was correlated with the significant increase in blood Lactate	11
Murawska et al. 2015	12 (7/5)	Physically active	25.6 ± 5.8	Serum	3 months/2 times a week/CrossFit, whole body workout combined with treadmill or cycling	60-min of WOD: 15-min strength training (dumbbells and bars) followed with 10-min whole body aerobic exercise circuit and finishing with 15-min of cycling or treadmill	Long-term, high-volume, Long-interval HIIT	Baseline BDNF increased after intervention, then lowered after progressive Wingate test in males but no changes in women	11
Nicolini et al. 2019	18 (18/0)	Sedentary	23.1 ±	Serum	6 weeks/3 sessions a week/cycle-ergometer	3-min warm up at 50% W_peak_, 5 × 60 s high bouts at 105–135% W_peak_ with 90 s active recovery at 30% W_peak_	Mid-term, low-volume, moderate-interval HIIT	Improvement of cardiorespiratory fitness but no significant changes in BDNF concentrations after 18 sessions of HIIT in sedentary individuals	8
Rentería et al. 2019	17 (0/17)	Sedentary	21.5 ± 1.6	Serum	4 weeks/3 times a week/cycle-ergometer	15- to 25-min of 3 to 5 30 s bouts of high intensity at 80% of MAP with 4-min of active recovery at 40% MAP	Mid-term, low-volume, moderate-interval	BDNF concentrations increased after intervention and lowered after graded-exercise test post-HIIT	7
Reycraft et al. 2019	8 (8/0)	Physically active	23.1 ± 3	Plasma	Crossover acute training/self-propelled treadmill	18-min, 4 × 30 s bouts of all-out running interspersed with 4 min of active recovery	Acute, low-volume, SIT	BDNF concentrations were increased immediately post SIT and recovered baseline concentrations 30 and 90 min after exercise, no incremental changes were observed in other modalities	7
Rodríguez et al. 2018	6 (6/0)	Physically active	22.6 ± 0.7	Serum	Acute high intensity exercise/motorized treadmill	5-min warm-up at 50–60%VO_2max_ followed by 4 4 min bouts at 85% VO_2max_ combined with 3-min active recovery at 40%VO_2max_	Acute/high-volume, long-interval HIIT	Significant BDNF increase after HIIE but the changes were not correlated with the increase in lactate	7
Sadowska et al. 2019	8 (8/0)	Physically active	23.1 ± 1.7	Serum	6 weeks/5 days a week/CrossFit combined with running track field	50-min of WOD composed with aerobic whole body circuit, aerobic training and weightlifting of predetermined sets or AMRAP	Mid-term, high-volume, combination of repeated sprint and SIT	Slight increase of BDNF levels after CrossFit training, but no changes after aerobic testing were performed	7
Saucedo-Marquez et al. 2015	21 (21/0)	Physically active	28 ±	Serum	Crossover acute training/cycle-ergometer	20-min of 60 s at 90% VO_2max_ with 60 s active rest period	Acute, moderate-volume, moderate-interval HIIT	Greater increase of peripheral BDNF in HIIT compared to CON, with a higher magnitude of change in contrast to baseline measures	11
Slusher et al. 2018	13 (13/0)	Sedentary	23.6 ± 1.0	Serum and Plasma	Acute high intensity exercise/cycle-ergometer	5-min, 20 s 170% VO_2peak_ at 5.5% bodyweight interspersed with 10 s active recovery	Acute, low-volume, SIT	Serum BDNF increased significantly after HIIE and remained higher following the completion of executive function test; otherwise plasma BDNF was not modified in either post-HIIE or after completion of executive function text	7

*Note:* AMRAP: As many repetitions as possible, BDNF: Brain-Derived Neurotrophic Factor. CON: Control group, HIIE: High Intensity Intermittent Exercise, HIIT: High Intensity Interval Training, MAP: Maximal aerobic power, P_max_: Maximal power, SIT: Sprint Interval Training, ST-HIIT: Short-term High Intensity Interval Training, _s_VO_2max_: Maximal speed reached during VO_2max_, VO_2max_: Maximal oxygen consumption, VO_2peak_: Peak oxygen consumption, W_max_: Maximal Wattage, WOD: Workout of day, W_peak_: Peak Wattage.

Therefore, the purpose of this systematic review and meta-analysis was to examine the effects of acute and chronic IT on circulating BDNF concentrations in apparently healthy young adults. In regard that the exercise response of BDNF is influenced by gender^41,42^, fitness level^43,44^, and exercise intensity^45–47^ an analysis of moderator variables by subgroups were performed. Finally, we assess the differences of changes in circulating BDNF between serum and plasma after IT.

## Methods

### Overview

This study followed the methodologies to complete a systematic review and meta-analysis suggested by Moher et al. (2015)^48^ and the International Prospective Register of Systematic Reviews (PROSPERO). The protocol was registered at PROSPERO under the code CRD42019122687.

### Eligibility criteria

Studies that met the following criteria were included: (1) randomized controlled trials (RCT) and controlled trials without randomization (pre-test), (2) healthy normal-weight participants (as determined by a body mass index (BMI) between 20 to 24 kg/m^2^ or a body fat mass < 20% for men and < 28% for women, (3) young adults (18 to 40 yr. old), (4) male and female of different ethnic groups, (5) interventional studies, (6) serum and plasma circulating BDNF, (7) studies including participants free of any pharmacological prescription medication or drug, or recreational smoking, (8) studies using the enzyme-linked immunosorbent assay (ELISA) method to determine circulating BDNF. Studies that met the following criteria were excluded: (1) studies involving overweight and obese participants (BMI > 25 kg/m^2^), (2) children and adolescents (< 18 years old), (3) middle age and elderly people (> 40 yr. old), (4) pregnant women, and (5) cross-sectional studies.

### Information sources

Seven electronic databases (PubMed, Science Direct Collection, Scopus, SpringerLink, Taylor & Francis journals, Wiley Online Library, Web of Science) were searched for potentially eligible studies in English. In addition, cross-referencing from retrieved studies was conducted. The last searches were conducted on February 2020 by two researchers (PCG-S and AJ-M).

### Search strategy

Search strategies were developed using text words as well as Medical Subject Headings associated with the effects of exercise on BDNF. The search strategy included the following key words in English language: interval training, BDNF, intermittent training, high intensity intermittent training, interval running, brain-derived neurotrophic factor, high-intensity interval training, HIIT, sprint interval training, SIT, CrossFit, Tabata. Boolean operators AND, OR, NOT OR Mesh option were used to concatenate the search terms (key words). A secondary search was performed by screening the reference list of the selected studies and relevant review articles. Finally, a forward citation tracking of the selected studies was conducted through Scopus. An example of the search strategy for one of the databases searched (PubMed) is shown in supplementary Fig S1 online.

### Study records and selection

All studies to potentially be screened were imported into Mendeley software, version 1.19.3 (Elsevier Inc., New York, NY, USA). One author then removed duplicates both electronically and manually. A copy of the database was then provided to two authors for duplicate screening. The two authors selected all studies, independent of each other. The full report for each article was obtained for all titles and abstracts that appeared to meet the inclusion criteria or where there was any uncertainty. Reasons for exclusion were coded as one or more of the following: (1) duplicates (2) missing or incomplete descriptive statistics (3) inappropriate research design (4) language different to English (5) abstracts only and (6) animal model. Upon completion, the two authors met and reviewed their selections. Given the small number of studies selected, discrepancies were reached by consensus. Based on the final number of studies to be included, the overall precision of the searches was calculated by dividing the number of studies included by the total number of studies screened after removing duplicates. The number needed to read (NNR) was then calculated as the inverse of the precision^49^.

### Data extraction

Titles and/or abstracts of studies retrieved using the search strategy and those from additional sources were screened independently by two review authors (PCGS and AJM) to identify studies that potentially met the inclusion criteria outlined above. The full text of these potentially eligible studies was retrieved and independently assessed for eligibility by two review team members. Any disagreement between them over the eligibility of particular studies was resolved through discussion with a third reviewer (IR).

The studies were retrieved in Mendeley software, version 1.19.3 (Elsevier Inc., New York, NY, USA) and exclusion reasons were recorded. Data were exported to a standardized, pre-piloted Excel spreadsheet used to extract data from the included studies for assessment of study quality and evidence synthesis. The extracted information included publication year, participant demographics and baseline characteristics (e.g., gender, age, cardiorespiratory fitness level), details of the intervention (e.g., exercise frequency, intensity, duration, session duration, total duration of the intervention, dropouts) and control conditions, outcomes (i.e., serum and plasma BDNF) (mean and standard deviation). Two review authors extracted data independently and discrepancies were identified and resolved through discussion with a third author. Missing data were requested from study authors.

### Primary outcome

The primary outcome was the change in peripheral BDNF concentration between control and experimental conditions (i.e., repeated measures design) or groups (i.e., independent group design). It is worth noting the first post-exercise BDNF measure was considered for analysis.

### Risk of bias assessment in individual studies

Two review authors independently assessed the risk of bias in included studies by using the Tool for the Assessment of Study Quality and Reporting in Exercise (TESTEX)^50^. The TESTEX is a 12-item (5 points for study quality and 7 points for reporting) and 15-point scale (5 points for study quality and 10 points for reporting) developed to facilitate a comprehensive review of exercise training trials. Disagreements between the review authors over the risk of bias in particular studies were resolved by discussion, with involvement of a third review author where necessary.

### Data synthesis and calculation of effect sizes

The effect size (ES) was calculated as the difference between means according to the methodology proposed by Borenstein, Hedges, Higgins, and Rothstein (2009)^51^. For the calculation, the initial score (pre-test) of BDNF was compared with the final score (post-test) after an intervention (exercise). The ES was subsequently adjusted to take into account the bias introduced by small samples^52^. For the analysis, the random effects model was used, which assumes that ESs vary between studies^51,53^. In this study, ES was interpreted as trivial (0 to 0.19), small (0.20 to 0.49), moderate (0.50 to 0.79) and large (≥ 0.80)^54^. ANOVA and independent samples t-test were used to determine mean ES differences between categorical moderator variables.

### Meta-biases

Small-study effects (publication bias, etc.) were assessed following current recommendations^55,56^. The degree of heterogeneity of the studies was analyzed through Cochran’s Q test^57^ and the degree of consistency between the studies was calculated through the *I*^2^ test^58^. The *I*^2^ statistic ranges from 0 to 100%, and is interpreted as low (≤ 25%), moderate (26–74%) and high (≥ 75%)^58^. The effect of the studies with small samples was determined by the Doi plot and LFK index^55^. LFK index values outside the interval between −1 and + 1 are considered consistent with asymmetry (i.e. publication bias)^59^. An α level ≤ 0.05% and 95% confidence intervals (95% CI) that did not include zero (0) were considered to represent statistically significant small-study effects.

### Software used for data synthesis

All data were analyzed using IBM SPSS Statistics for Windows, Version 23.0 (Armonk, NY), Microsoft Excel V.2010 and the Meta XL V.5.3, 2016 add-in software for Excel (EpiGear Intl., Queensland, Australia).

## Results

### Study characteristics

**Figure 1:**
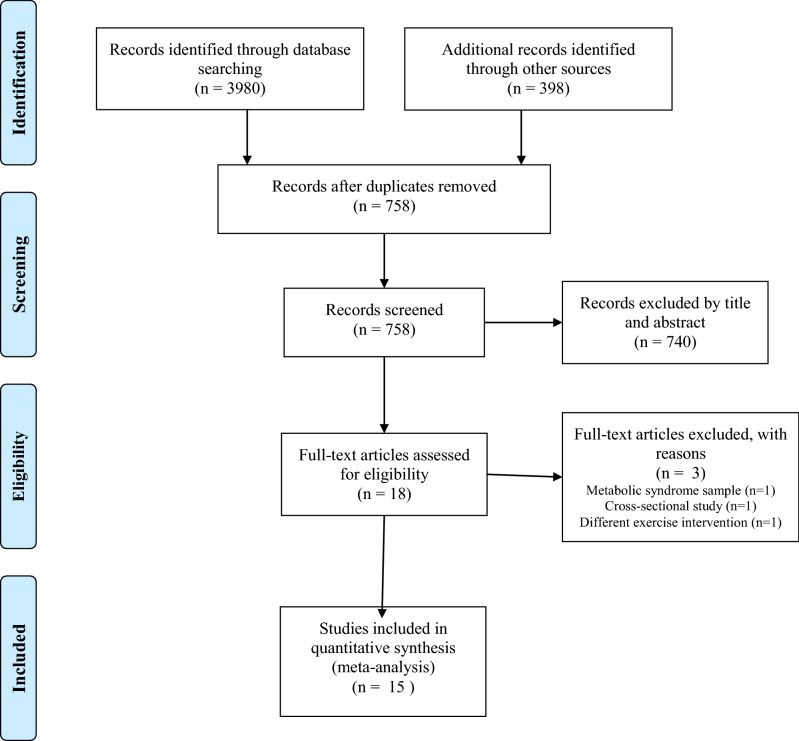
Flow diagram depicting the search process.

A flow diagram that depicts the search process for study selection is shown in Fig. 1. After initially identifying 4378 citations and removing 3620 duplicates both electronically and manually, 758 citations were screened. Of these, 18 studies met the criteria for inclusion, and three studies were excluded (one study used a sample with metabolic syndrome, one study used a cross-sectional design, and one study used a different exercise intervention). The major reasons for exclusion were: (1) duplicates (82.7%), (2) title and abstract did not meet the test subjects’ inclusion criteria (16.9%), (3) Cross-sectional study and other modalities different from IT (0.7%). The precision of the search, excluding duplicates, was 2% while the NNR was 51. Twenty-two ESs were computed from 15 studies representing 277 participants meeting the criteria for inclusion (Table 1).

### Participant and exercise characteristics

The mean age of the participants was 24.8 ± 4.4 yr., and the mean number of participants in the studies was 19 ± 15 participants, with most studies recruiting males (67%), mixed samples (20%), and a small number of studies recruiting females (13%). Participants in the studies were physically active (60%), sedentary (33%), and athletes (7%). Nine studies (60%) recorded acute exercise responses and six studies (40%) recorded chronic training effects. Specific types of activities included IT on a cycle-ergometer (54%), treadmill (27%), and combined (20%), including whole-body circuits, CrossFit, and running on a track field (Table 1). Overall, five studies assessed BDNF response on HIIT (34%) and ten studies in SIT (66%).

### Risk of bias assessment

Results for risk of bias assessment using the TESTEX scale showed that overall, studies achieved 54.7% of the quality requirements. Therefore, 45.3% of the studies were at an unclear or high risk of bias concerning: (1) eligibility criteria specified (100%), (2) randomization specified (53%), (3) allocation concealment (53%), (4) groups similar at baseline (53%), (5) blinding of assessor (0%), (6) outcome measures assessed in 85% of patients (13%), (7) intention-to-treat analysis (73%), (8) between-groups statistical comparisons reported (50%), (9) point measures and measures of variability for all reported outcome measures (80%), (10) activity monitoring in control groups (73%), (11) relative exercise intensity remained constant (10%), and (12) exercise volume and energy expenditure (93%). Given the inability to truly blind participants in exercise intervention trials, all studies (100%) were considered to be at a high risk of bias for the categories “allocation concealment” and “blinding of assessor”. In addition, 87% of the studies did not report adverse effects and 13% of the studies reported adherence to exercise interventions.

### Data synthesis

**Figure 2:**
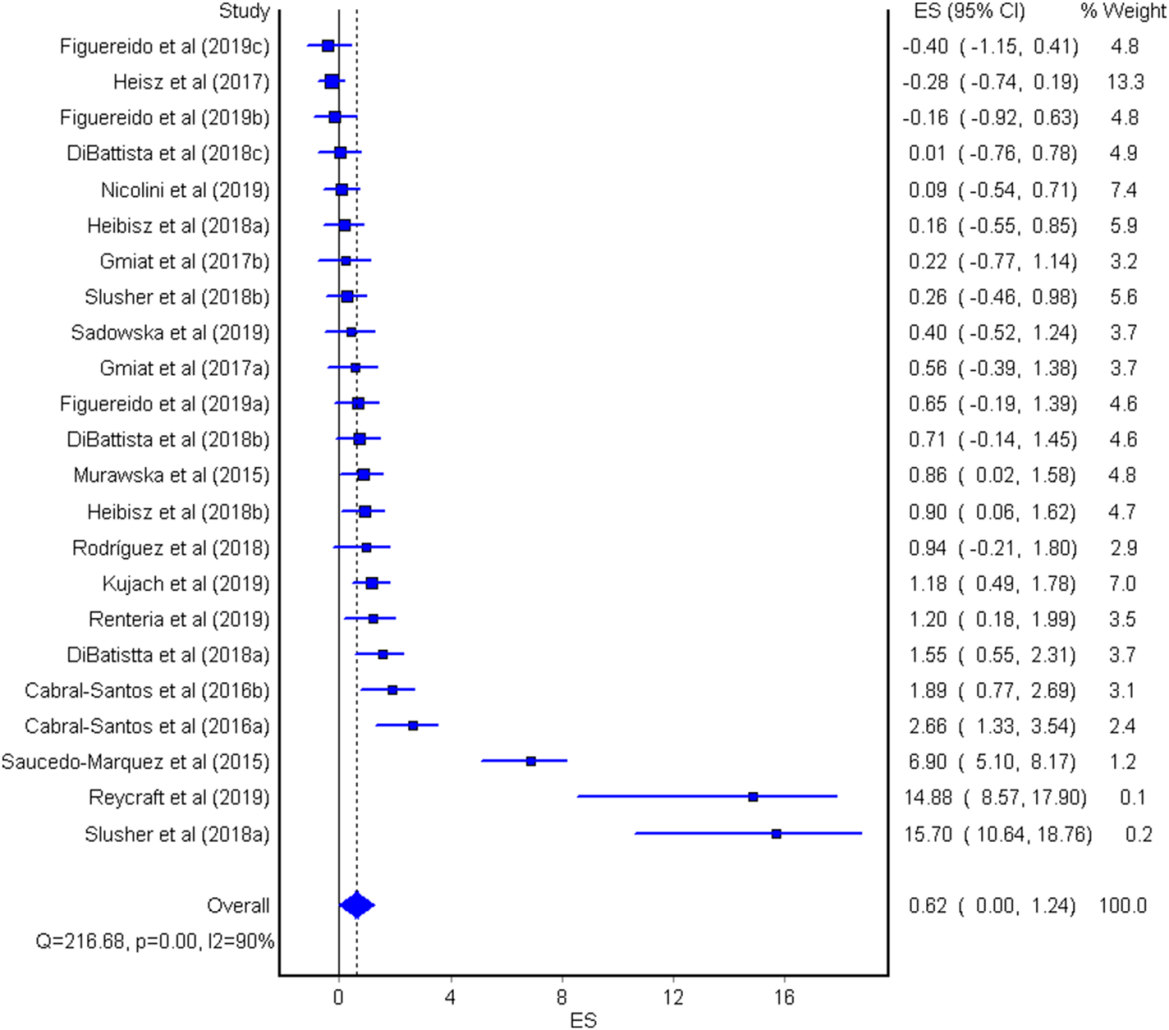
The overall effect of interval training (IT) on peripheral BDNF concentration. The lines indicate 95% confidence intervals (CI), and the square reflects the standardized differences (SMD) for each study. The diamond in the forest plot indicates the overall effect size (ES).

The overall effect of IT on peripheral circulating BDNF concentrations was moderate and significant (ES = 0.62, 95% CI = 0.00, 1.24, Fig. 2). The studies provided heterogeneous results (Q = 216.68, p < 0.001), showed high inconsistency (*I*^2^ = 90%), and major asymmetry (LFK index = 2.76, see Supplementary Fig. S2 online).

**Figure 3:**
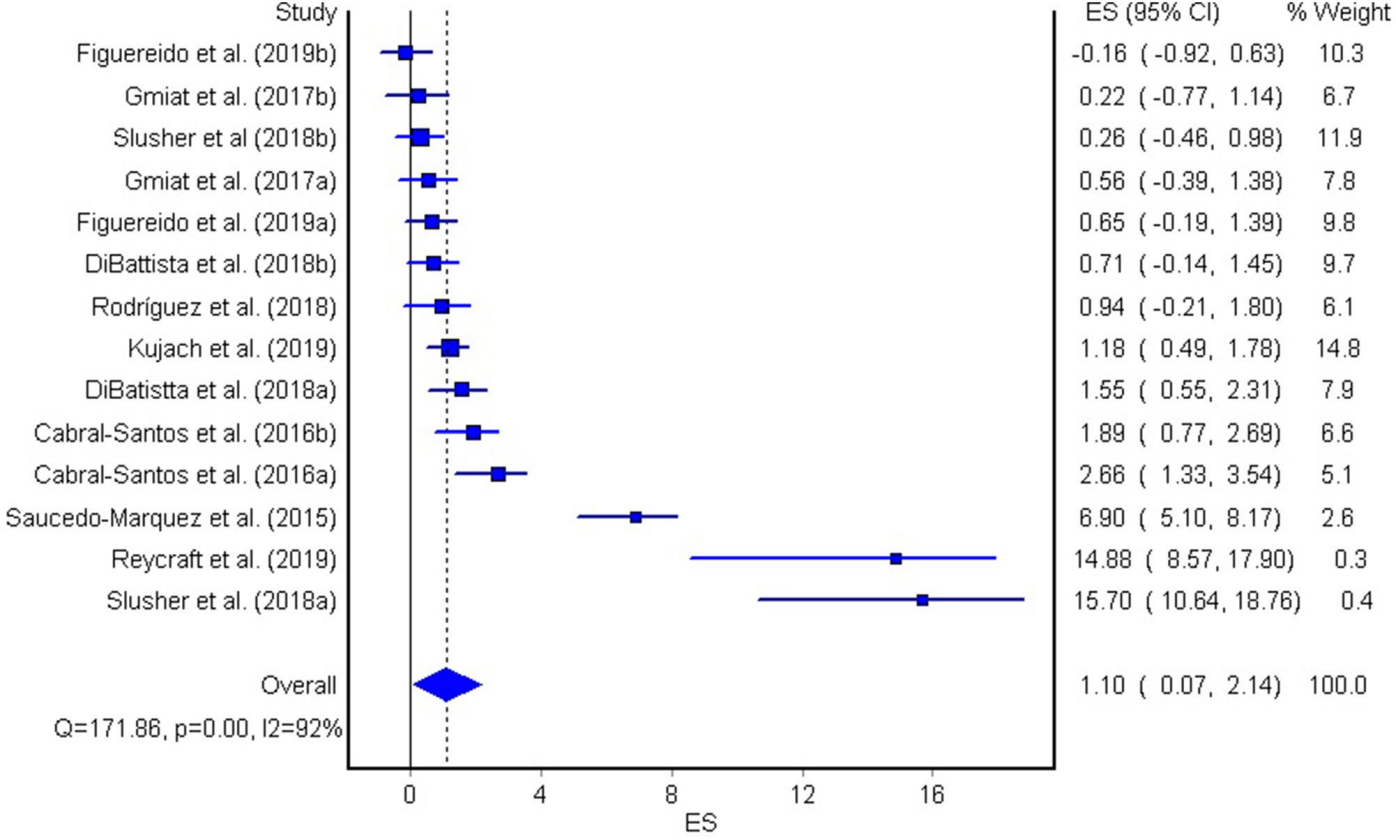
The effect of acute interval training (IT) on circulating BDNF concentration. The lines indicate 95% confidence intervals (CI), and the square reflect the standardized differences (SMD) for each study. The diamond in the forest plot indicates the overall effect size (ES).

**Figure 4:**
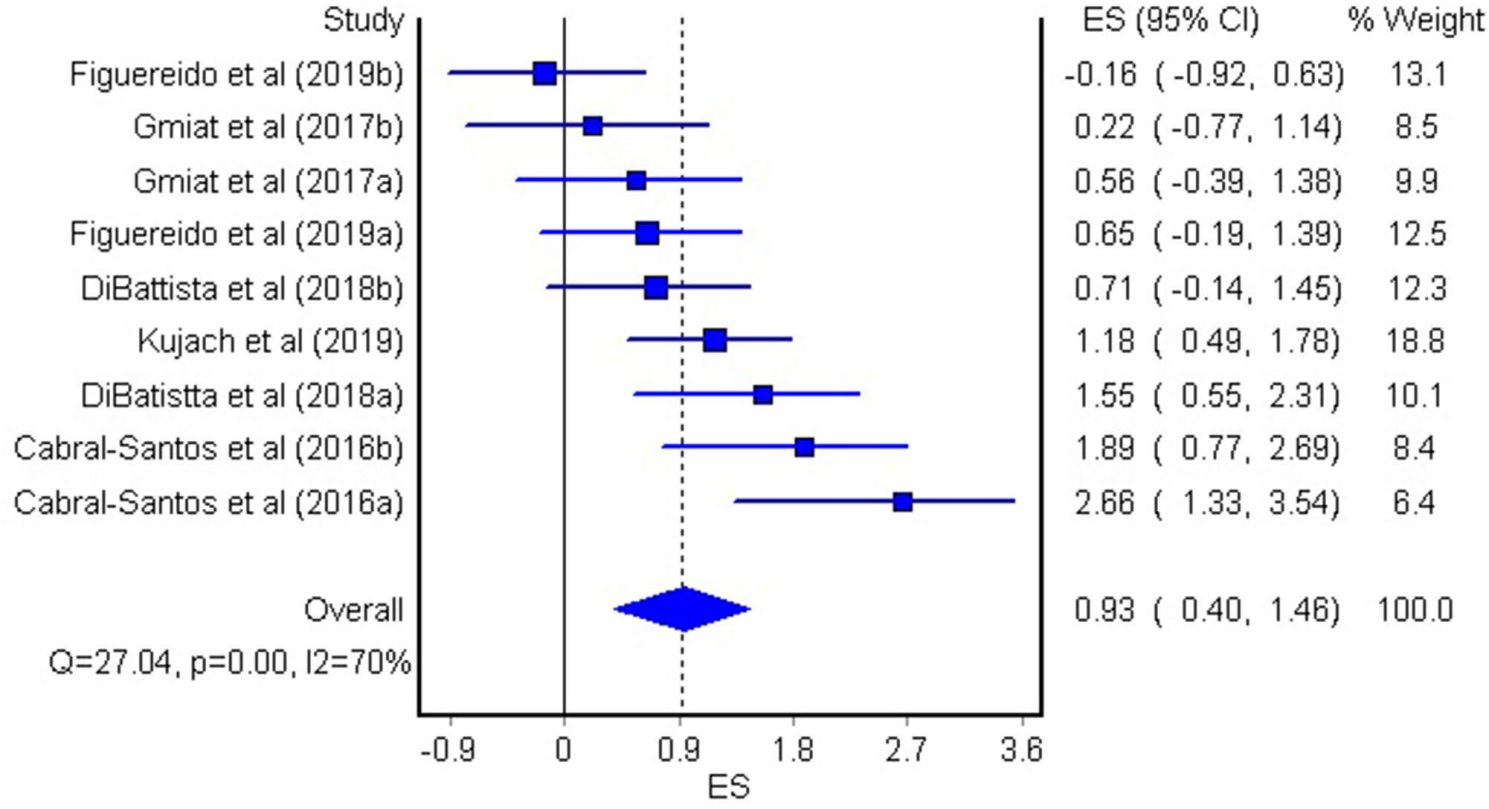
The effect of chronic interval training (IT) on circulating BDNF concentration. The lines indicate 95% confidence intervals (CI), and the square reflects the standardized differences (SMD) for each study. The diamond in the forest plot indicates the overall effect size (ES).

The acute effect of IT on circulating BDNF was large and significant (ES = 1.10, 95% CI = 0.07, 2.14, Fig. 3). However, similar to the overall effect, the studies provided heterogeneous results (Q = 171.86, p < 0.001), and showed high inconsistency (*I*^2^ = 92%), and major asymmetry (LFK index = 3.34, see Supplementary Fig. S3 online). The chronic effect of IT on BDNF was large and significant (ES = 0.93, 95% CI = 0.40, 1.46, Fig. 4). The studies provided heterogeneous results (Q = 27.04, p < 0.001), showed moderate inconsistency (*I*^2^ = 70%), and minor asymmetry (LFK index = 1.21, see Supplementary Fig. S4 online).

Categorical moderator variable analysis on acute and chronic exercise interventions showed that there is no statistically significant subgroup effect for fitness level, type of training, and medium (serum vs. plasma) for acute and chronic IT (Table 2). There was a strong tendency (p = 0.052) for gender in chronic IT analysis; a higher ES was observed in females during chronic IT intervention compared with males (Table 2). For continuous moderators, no significant correlations were found between ES and age for acute (r = -0.18, p = 0.534) and chronic (r = -0.09, p = 0.805) exercise. No significant correlations were found between ES and sample size for acute (r = -0.20, p = 0.501) and chronic (r = -0.45, p = 0.192) exercise.

**Table 2:** Moderator variables for the effect of acute and chronic exercise on BDNF.

Variable	n = studies	ES ± SD	95%CI		p =
			Lower-limit	Upper-limit	
**Acute exercise**					
Gender					0.405
Male	12	3.93 ± 5.61	0.76	7.10	
Female	2	0.39 ± 0.24	0.06	0.72	
Fitness					
Sedentary	4	1.19 ± 7.68	− 6.33	8.71	0.750
Active	10	3.12 ± 4.57	0.29	5.95	
Type of training					0.824
HIIT	8	3.13 ± 5.57	− 0.74	7.00	
SIT	6	3.81 ± 5.46	− 0.56	8.18	
Blood analysis					0.698
Plasma	4	4.35 ± 7.04	− 2.55	11.3	
Serum	10	3.05 ± 4.88	− 0.44	6.55	
**Chronic exercise**					
Gender					
Male	5	0.15 ± 0.40	− 0.35	0.65	0.052
Female	2	1.25 ± 0.06	0.67	1.82	
Mixed	3	0.26 ± 0.60	− 1.22	1.74	
Fitness					0.950
Sedentary	3	0.34 ± 0.77	− 1.58	2.25	
Active	5	0.39 ± 0.64	− 0.41	1.19	
Athlete	2	0.53 ± 0.52	− 4.17	5.23	
Type of training					0.208
HIIT	4	0.48 ± 0.69	− 0.62	1.57	
SIT	3	− 0.08 ± 0.29	− 0.80	0.64	
CrossFit	3	0.78 ± 0.46	− 0.36	1.92	

## Discussion

The present study was designed to systematically-review and meta-analyze the effects of acute and chronic IT on circulating BDNF concentration in young adults. Overall, acute and chronic IT increased peripheral BDNF concentration. In chronic IT, females showed greater increases in BDNF compared with males. Finally, the study showed that the fitness levels did not regulate the BDNF response after IT, at least in the studied population (apparently healthy young adults).

The data of the current study are in agreement with the previous report focused on the impact of aerobic exercise on peripheral BDNF^60^. In Dinoff’s study, the exercise protocols were longer than the interventions analyzed in the current meta-analysis (≤ 30 min/session). This finding suggests that IT is an effective treatment to improve brain health with more time efficiency than MICT. The latter condition is concordant with peripheral adaptations induced by IT (e.g., oxidative capacity in muscle, cardiometabolic markers)^31,37,61^.

IT is characterized by lactate accumulation in blood^45,62–64^. Studies in rodents have demonstrated that blood lactate (BLa) produced during exercise reaches the brain and enhances expression of genes associated with cognition (i.e. *Bdnf*)^65,66^. Although in humans this response has not been completely demonstrated, authors suggested a similar effect of BLa in brain^45,63,67,68^. Resulting in diverse improvements in executive function^63^. Unfortunately, in the current meta-analysis, there were not enough studies that reported blood lactate changes; consequently, it was not possible to run meta-regressions to identify the role of this metabolite in the BDNF response.

Non-statistical differences were found among BDNF changes in plasma and serum (Table 2). While some studies did not find statistical differences between BDNF changes in serum and plasma following physical exercise^27^, others reported significant changes in circulating BDNF in plasma compared with serum^60^. In previous studies, aerobic, strength, and concurrent training were analyzed, whereas, in the current meta-analysis, IT interventions were examined. Circulating BDNF changes are sensitive to training modality^45,69^; therefore, it was not possible to compare our data with other systematic and meta-analytic works^27,60^.

In serum, BDNF concentration is > 50 fold higher than plasma^18,70,71^. In the periphery, platelets store BDNF; therefore, these cells are considered the major reservoir of circulating BDNF^71,72^. Once activated, platelets release BDNF^18,71^. This process is considered the main mechanism to explain differences between serum and plasma concentrations^73,74^. The evidence suggests that chronic training improves the capacity of platelets to release BDNF^18,75^. Concerning this, we did not discard that the length of interventions examined in the current study was insufficient to modify the platelet’s capacity in the BDNF secretion; thus, further studies are necessary to elucidate this hypothesis. In addition, it is known that IT is an exercise modality that increases muscle damage^76^. We believe that this condition could be present in the participants and consequently will generate platelet activation^71^, releasing BDNF to repair muscle injuries^77^. This physiological response might explain the lack of differences among the BDNF changes in serum and plasma (Table 2). Finally, we did not discard that the small numbers of studies included in the current meta-analysys can explain the lack of differences among the blood mediums.

Furthermore, it is worth noting that plasma volume (PV) changes should be considered in studies that assess the impact of exercise on biomarkers such as neurotrophins. It is known that exercise modifies PV^78^, which can increase biomarker concentrations. Thus, the results of studies neglecting to measure PV changes should be viewed with caution^78–80^. In one study examined, there was no effect on circulating BDNF with IT when PV was not adjusted. In contrast, DiBatista et al. showed that IT increased BDNF levels following PV adjustment. Finally, in work conducted by Reycraft and colleagues,PV was not adjusted and the authors reported a significant effect of IT on BDNF. The results of these studies show that PV should be considered when evaluating the effects of IT on circulating BDNF levels. Moreover, studies where BDNF was assessed in plasma were fewer than studies where the biomarker was measured in serum. Thus, unequal distribution can be a confounding variable to find statistical differences.

Similarly, to the medium, fitness level did not significantly affect the BDNF response to IT. These findings are contrary to previous reports^18,44,60,75,81^. Despite the established negative correlation between fitness level and BDNF response during exhaustive or aerobic exercise^44,60^, biochemical and physiological mechanisms are not fully understood. One hypothesis suggests that well-trained participants have higher BDNF receptor levels in peripheral organs (e.g., skeletal muscle) which could attenuate circulating BDNF changes during exercise^82^. Once it activates the peripheral TrkB receptor, BDNF participates in the repair of skeletal muscle^77^. As indicated above, IT induces muscle damage in well-trained and untrained participants^76^; therefore, we did not discard that the low peripheral BDNF levels were induced by muscular damage after IT. That condition could partially explain the lack of significant differences in BDNF changes between athletes and untrained participants (Table 2). Another hypothesis suggests that trained participants show better cognitive performance than sedentary people^44,83^. Indeed, athletes and well-trained individuals have more efficient uptake and utilization of BDNF which has been shown to improve neural plasticity and improve performance in cognitive tasks compared to untrained participants^44,83^. The extensive utilization of BDNF in brain reflects a lower peripheral BDNF in athletes and well-trained participants with respect to untrained people^44,83^. Therefore, we do not discard the possibility that active participants show a high capacity to uptake BDNF in brain after IT compared with sedentary participants (Table 2). In contrast, sedentary participants have lower synthesis and release of BDNF. Both conditions combined resulted in a non-significant statistical effect among active and sedentary (Table 2).

The null findings observed in sedentary and active participants after acute IT can be explained by stress hormone activity. Specifically, IT is perceived as difficult and vigorous in well-trained and untrained population^45,76,84–86^. In agreement with this, IT increases systemic cortisol concentrations in athletes and untrained participants^47,87–89^. Cortisol is a hormone that decreases BDNF synthesis^90^. Therefore, higher cortisol levels could be present in the participant (sedentary, active, and athlete participants) after IT, reducing differences in BDNF changes (Table 2). Finally, we do not exclude the possibility that the small numbers of studies included in the current meta-analyses can explain the lack of differences among fitness levels.

We found a high ES (strong tendency) for females compared with males; a difference shown principally in chronic IT (Table 2). This may be explained by the role of steroid hormones since it is known the positive effect of 17β estradiol on BDNF synthesis in the brain^91–95^. The estrogen hormone concentrations change during the menstrual cycle^96^; particularly, high levels of estrogen are found during the late follicular phase^97^. In the studies analyzed in the current meta-analysis, the menstrual cycle was not coded; therefore, we do not discard the possibility that some of the blood collection made in females was performed during the follicular phase, resulting in an enhancement effect of estrogen to IT impact on BDNF changes compared to males. Additionally, as discussed previously, platelets store and release BDNF^71,72^. In this sense, classic and emerging studies show that women have higher platelet content than men^98–100^. In light of this, we do not discard that platelet count could contribute to a higher BDNF response in women compared with men (Table 2). Additionally, authors have previously suggested that skeletal muscle uptake BDNF; once captured the neurotrophin regulates metabolic and neuromuscular responses^101–103^. In females, muscle mass is lower than males^104–106^. Therefore, it is possible that differences skeletal muscle mass among sex, can explain the larger ES in women compared with men (Table 2).

**Figure 5:**
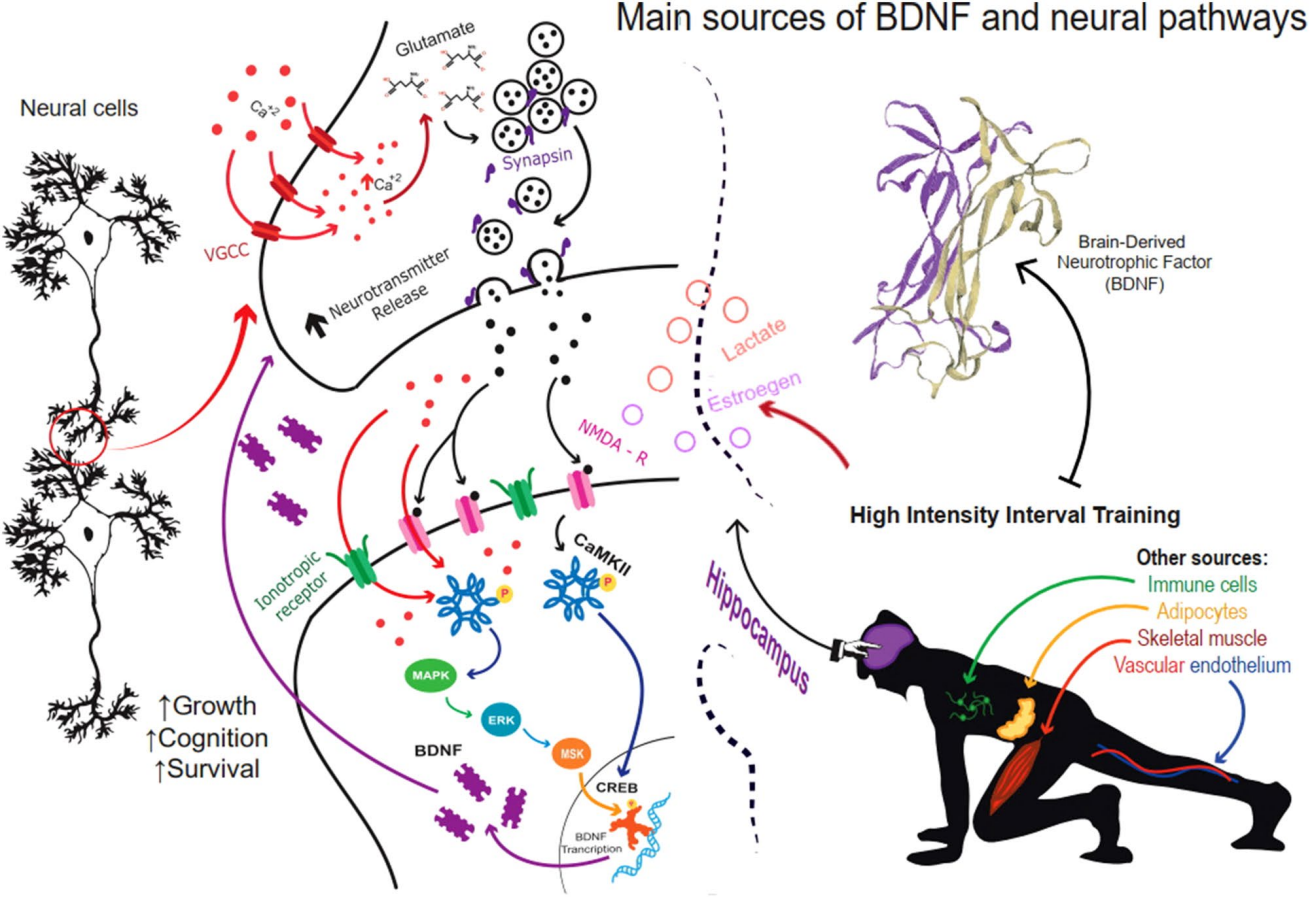
Interval training increases circulating BDNF levels in healthy adults (upper right). During this response, the brain (hippocampal region) seems be the main BDNF source; nevertheless, other tissues function as BDNF synthesizers. The mechanism of activation during IT has not elucidated yet (above right). In brain, BDNF synthesis is activated by an increase of calcium (Ca2+) concentrations in the cytosol. Inside neurons, Ca2+ activates calmodulin dependent kinase II (CaMKII), triggering activation of the MAPK/ERK/MSK cascade resulting in an increase in the expression and phosphorylation of cAMP response element-binding protein (CREB). CREB initiates BDNF transcription resulting in increased BDNF synthesis and release (left). Once secreted, the neurotrophin regulates molecular mechanisms associated with neuronal growth, cognition, and neuron survival (above left). Finally, scientific evidence suggests that other circulating molecules such as lactate and estrogen enhance BDNF synthesis in brain (center). The putative mechanism indicate that lactate increases calcium current in the neurons, and estrogens activates nuclear estrogen receptors and membrane estrogen receptors that enhance the BDNF synthesis. Figure made with adobe illustrator cs6. https://www.adobe.com/products/illustrator/free-trial-download.html. Figure conceived and designed for PCGS.

The current meta-analysis highlights that IT is an effective strategy to increase peripheral BDNF concentrations in young healthy adults. Our findings are in agreement with prior meta-analysis focused on assessing the impact of physical exercise (e.g., aerobic and strength exercise) on circulating BDNF in young adult and healthy population^27,60,107^ (Fig. 5). This finding adds relevant information to previous studies reporting a positive impact of IT on fitness levels^108–110^, and hemodynamic variablesy^111^. Therefore, the state of the art, based on quantitative analysis suggests that IT may be considered an adequate physical exercise modality to strengthen the health (brain and peripheral physiological functions) in an apparently healthy young adult population.

## Supplementary Information

Supplementary Information

Supplementary Figure 1

Supplementary Figure 2

Supplementary Figure 3

Supplementary Figure 4

## Data Availability

The data that support the findings of this study are available from the corresponding author on request.
