# Supplementary material for: The effects of interval training on peripheral brain derived neurotrophic factor (BDNF) in young adults: a systematic review and meta-analysis
Source: Sci Rep. 2021 Apr 26;11:8937. doi: 10.1038/s41598-021-88496-x (PMC8076263; doi:10.1038/s41598-021-88496-x)
Supplement: Supplementary file 1 — Supplementary Information [file 41598_2021_88496_MOESM1_ESM.doc]

**The effects of interval training on peripheral brain derived neurotrophic factor (BDNF) in young adults: a systematic review and meta-analysis**

Patricia Concepción García-Suárez^1^, Iván Rentería^1^, Eric P. Plaisance^2^, José Moncada-Jiménez^3^

Alberto Jiménez-Maldonado^1^*

^1^Facultad de Deportes Campus Ensenada, Universidad Autónoma de Baja California, Ensenada, 22890, México.
^2^Department of Human Studies, University of Alabama at Birmingham, Birmingham, 35294, United States of America.
^3^Human Movement Sciences Research Center, University of Costa Rica, San José, 1200, Costa Rica

**Supplementary figure legends**

**Supplementary Figure 1**. PubMed search example

**Supplementary Figure 2.** The symmetry of overall BDNF results is presented in the Doi plot

**Supplementary Figure 3.** The symmetry of acute BDNF results is presented in the Doi plot

**Supplementary Figure 4.** The symmetry of chronic BDNF results is presented in the Doi plot
